# Lipophosphoglycan polymorphisms do not affect *Leishmania amazonensis* development in the permissive vectors *Lutzomyia migonei* and *Lutzomyia longipalpis*

**DOI:** 10.1186/s13071-017-2568-8

**Published:** 2017-12-16

**Authors:** Paula M. Nogueira, Agna C. Guimarães, Rafael R. Assis, Jovana Sadlova, Jitka Myskova, Katerina Pruzinova, Jana Hlavackova, Salvatore J. Turco, Ana C. Torrecilhas, Petr Volf, Rodrigo P. Soares

**Affiliations:** 1 Instituto René Rachou/FIOCRUZ, Belo Horizonte, MG Brazil; 2 Departamento de Parasitologia, UFMG, Belo Horizonte, MG Brazil; 3 Department of Parasitology, Faculty of Science, Charles University, Prague, Czech Republic; 4 Department of Biochemistry, University of Kentucky Medical Center, Lexington, KY USA; 5 Laboratório de Imunologia Celular e Bioquímica de Fungos e Protozoários, Departamento de Farmácia, UNIFESP, São Paulo, SP Brazil

**Keywords:** *Leishmania amazonensis*, Lipophosphoglycan, *Lutzomyia longipalpis*, *Lutzomyia migonei*, *Phlebotomus papatasi*, Vector-parasite interaction

## Abstract

**Background:**

Lipophosphoglycan (LPG) is a dominant surface molecule of *Leishmania* promastigotes. Its species-specific polymorphisms are found mainly in the sugars that branch off the conserved Gal(β1,4)Man(α1)-PO_4_ backbone of repeat units. *Leishmania amazonensis* is one of the most important species causing human cutaneous leishmaniasis in the New World. Here, we describe LPG intraspecific polymorphisms in two *Le. amazonensis* reference strains and their role during the development in three sand fly species.

**Results:**

Strains isolated from *Lutzomyia flaviscutellata* (PH8) and from a human patient (Josefa) displayed structural polymorphism in the LPG repeat units, possessing side chains with 1 and 2 β-glucose or 1 to 3 β-galactose, respectively. Both strains successfully infected permissive vectors *Lutzomyia longipalpis* and *Lutzomyia migonei* and could colonize their stomodeal valve and differentiate into metacyclic forms. Despite bearing terminal galactose residues on LPG, Josefa could not sustain infection in the restrictive vector *Phlebotomus papatasi*.

**Conclusions:**

LPG polymorphisms did not affect the ability of *Le. amazonensis* to develop late-stage infections in permissive vectors. However, the non-establishment of infection in *Ph. papatasi* by Josefa strain suggested other LPG-independent factors in this restrictive vector.

## Background

*Leishmania amazonensis* is the causative agent of localized cutaneous leishmaniasis (LCL) and anergic diffuse cutaneous leishmaniasis (ADCL). This species, found in the Amazon basin, is transmitted by *Lutzomyia flaviscutellata* (Diptera: Psychodidae), widely distributed in South America [1, 2].

The surface of *Leishmania* spp. is covered by several glycosylphosphatidylinositol (GPI)-anchored molecules. Those glycoconjugates are important for parasite survival either in vertebrate or invertebrate hosts. Among these, the most studied is lipophosphoglycan (LPG), a multivirulence factor covering the entire protozoan surface and flagellum. Structurally, LPGs have four domains: a conserved glycan core region of Gal(α1,6)Gal(α1,3)Gal_f_(β1,3)[Glc(α1)-PO_4_]Man(α1,3)Man(α1,4)-GlcN(α1) linked to a 1-*O*-alkyl-2-*lyso*-phosphatidylinositol anchor, a region of repeat units Gal(β1,4)Man(α1)-PO_4_ and a small oligosaccharide cap [3].

Several biochemical analyses of LPG revealed intra and inter-species polymorphisms in the sequence and composition of sugars attached to repeat units. Early studies determined that these interspecies variations were important for attachment in the vector and for virulence in the vertebrate hosts [3, 4]. In the Old World species, intraspecific LPG variations were reported for *Leishmania major* [5, 6], *Leishmania tropica* [7, 8] and *Leishmania donovani* [9] and their ability to infect sand fly species (*Phlebotomus papatasi*, *Phlebotomus sergenti/Phlebotomus arabicus* and *Phlebotomus argentipes*) has been reported. In the New World, purified LPG from 14 strains of *Leishmania infantum* displayed important LPG polymorphisms (type I: no side-chains branching-off the repeat units; type II: one β-glucose residue branching-off the repeat units and; type III: 1–3 β-glucose as side-chains). However, those LPG variations did not affect the interaction with *Lutzomyia longipalpis* [10, 11]*.* Our preliminary data suggested qualitative LPG polymorphisms between two *Le. amazonensis* strains (PH8 and Josefa) based on antibody recognition, but they did not result in different activation in macrophages and/or sand fly infection [12].

Sand fly species can be divided into restrictive and permissive vectors depending on their ability to support development of various *Leishmania* species [13]. For example, *Ph. papatasi*, the restrictive vector, can sustain infection only with *Le. major* [4] and *Leishmania turanica* [14], two parasites with LPG terminated by β-galactosyl residues [15–17]. On the other hand, permissive vectors, like *Lu. longipalpis* and *Lutzomyia migonei*, can sustain infection of several *Leishmania* species; the former supports the development of *Le. infantum*, *Le. amazonensis* and *Le. major* (reviewed in [18]), whereas the latter supports the infection of *Le. amazonensis*, *Leishmania braziliensis* and *Le. infantum* [12, 19, 20].

The attachment of the parasite to a midgut receptor is a crucial event to avoid passage with the digested blood meal. The mechanisms of midgut attachment in restrictive vectors is LPG-dependent [21] and involves midgut galectin [22], while in permissive vectors it could be LPG-independent [23, 24] or may involve midgut mucin-like proteins [25].

The main vector of *Le. amazonensis* is *Lu. flaviscutellata.* However, established laboratory colonies of this species are not available. For this reason, *Lu. migonei* has been used as a successful model for interaction with *Le. amazonensis* [19]. Preliminary studies using *Le. amazonensis* strains (PH8 and Josefa) showed that they could survive for several days inside *Lu. migonei* [12]. However, their ability to reach the stomodeal valve and accomplish metacyclogenesis was not evaluated.

As a part of a wider project on the glycobiology of New World species of *Leishmania*, we described a detailed biochemical characterization of *Le. amazonensis* LPGs and found intraspecific differences. The interaction of *Le. amazonensis* strains with permissive and restrictive vectors was performed. Additionally, the ability of the Josefa strain, bearing terminal galactose residues in its LPG, to survive in the restrictive vector *Ph. papatasi* was evaluated.

## Methods

### Parasites, LPG extraction and purification

**Fig. 1:**
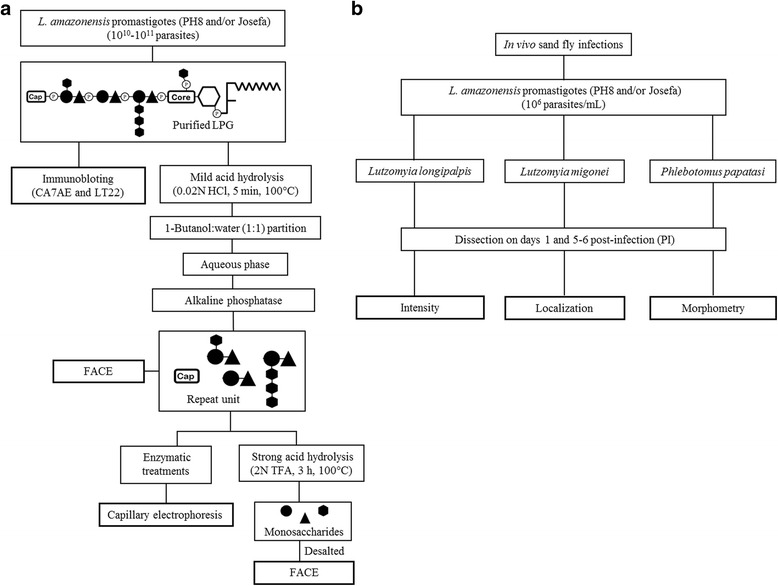
Procedures for the characterization of *Le. amazonensis* LPG repeat units and sand fly infections. **a** Purified LPGs were subjected to mild acid hydrolysis to depolymerize the repeat units and cap structures. Water-soluble fractions were partitioned using 1-butanol, treated with alkaline phosphatase (15 mM Tris buffer, pH 9.0, 1 unit, 16 h, 37 °C) and desalted by passage through a two-layered column of AG50W-X12 over AG1-X8. The desalted repeat units were subject to FACE analysis and enzymatic treatments with β-glucosidase, β-galactosidase to CE analysis. Additionally, the repeat units were subjected to strong acid hydrolysis (2 M trifluoroacetic acid, 3 h, 100 °C) and FACE assays to access monosaccharide composition. **b** Six combinations of *Le. amazonensis* strains (PH8 and Josefa) with *Lu. longipalpis*, *Lu. migonei* and *Ph. papatasi* were performed. Those were evaluated on days 1 and 5–6 post-infection (PI) for intensity, localization and morphometry

The Brazilian *Le. amazonensis* reference strain (IFLA/BR/1967/PH8) was isolated from the sand fly *Lu. flaviscutellata* from Pará State, whereas the Josefa strain (MHOM/BR/1975/Josefa) was isolated from a patient from Bahia State, Brazil. Molecular PCR identification was performed using primers for the HSP70 gene and kDNA minicircle [26, 27]. For immunoblotting, *Le. donovani* strain LD4 from Sudan (MHOM/SD/00/1S-2D) and the *Le. donovani* Mongi strain from India (MHOM/IN/1983/Mongi-142) were used as controls [9]*.* The strategy for purification and characterization of repeat units is depicted in Fig. 1a. Promastigotes were cultured in M199 medium supplemented with 10% fetal bovine serum (FBS). After the 6th day, stationary parasites were subjected to LPG extraction and purification as described elsewhere [28]. Purified LPGs (5 μg) were resolved by SDS-PAGE electrophoresis and transferred to nitrocellulose membrane. Blots were probed with monoclonal antibody (mAb) CA7AE (1:1000), that recognizes the unsubstituted Gal(β1,4)Man repeat units [29] and LT22 (1:1000) that recognizes β-glucose/β-galactose side chains [30]. After washing in PBS (3 × 5 min), the membrane was incubated for 1 h with anti-mouse IgG conjugated with peroxidase (1:10,000) and the reaction was visualized using luminol substrate [31].

### Fluorophore-assisted carbohydrate electrophoresis (FACE) of polysaccharides and mono and capillary electrophoresis (CE)

LPGs were subjected to mild acid hydrolysis (0.02 M HCl, 5 min, 100 °C) to depolymerize the repeat units and cap structures (Fig. 1a). Water-soluble fractions were partitioned using 1-butanol and repeat units were purified as previously described [10]. The neutral oligosaccharides from PH8 and Josefa repeat units were labeled with 0.05 M ANTS (8-aminonaphthalene-1,3,6-trisulfate) fluorophore and 1 M cyanoborohydride (37 °C, 16 h). Oligoglucose ladders (G_1_-G_7_) were used as standards. Sugars were subjected to FACE and the gel was visualized by a UV imager [7]. To confirm the sugars branching-off the repeat units and the linkages, they were treated with *E. coli* β-galactosidase in 80 mM Na_3_PO_4_, (pH 7.3, 4 U, 16 h, 37 °C) and sweet almond β-glucosidase in 200 mM ammonium acetate buffer (pH 5.0, 1 U, 16 h, 37 °C) [31]. Samples were labeled with 0.02 M APTS (8-aminopyrene-1,3,6-trisulfonic acid trisodium salt) in 15% acetic acid in sodium cyanoborohydride buffer and incubated overnight at 37 °C. Samples were run on CE at 25 kV for 20 min using reverse phase chromatography started with 10 s and 5 psi pressure injection [11].

To access monosaccharide composition, LPGs from both strains were subjected to strong acid hydrolysis (2 M trifluoroacetic acid, 3 h, 100 °C) (Fig. 1a). Depolymerized and desalted monosaccharides were fluorescently labeled with 0.1 M AMAC (2-aminoacridone) in 5% acetic acid and 1 M cyanoborohydride. Labeled sugars were subjected to FACE and the gel was visualized under UV light. Monosaccharides (D-galactose, D-glucose and D-mannose) (Sigma, St. Louis, MO, USA) were used as standards [32].

### Sand fly colonies

The *Lu. migonei* and *Lu. longipalpis* sand flies were initially captured from the Brazilian cities of Baturité (04°19′41"S, 38°53′05"W), Ceará State, and Jacobina (11°10′50"S, 40°31′06"W), Bahia State, respectively. *Phlebotomus papatasi* originate from South East Turkey. Laboratory colonies of the three sand fly species were maintained at Charles University, Prague, Czech Republic as previously described [33].

### Experimental infections of sand flies

Sand fly females were infected through the chick-skin membrane on a mixture of promastigotes and heat-inactivated rabbit blood; the final concentration of parasites was 1 × 10^6^ promastigotes/ml. The experiments were conducted with six sand fly-*Leishmania* combinations as depicted in Fig. 1b: *Lu. migonei*-PH8, *Lu. migonei*-Josefa, *Lu. longipalpis*-PH8 and *Lu. longipalpis*-Josefa, *Ph. papatasi*-PH8 and *Ph. papatasi*-Josefa. Terminal β-galactosyl residues were determinant for *Le. major* attachment to PpGalec in *Ph. papatasi* [22]. Since those sugars were also present in Josefa LPG, the ability of this strain to sustain infection in this vector was also checked compared to glucose-containing LPG from the PH8 strain.

Blood-engorged females were separated, maintained at 26 °C and dissected on days 1 and 5–6 post-infection (PI). Individual guts were placed into a drop of saline and examined microscopically for the localization and intensity of *Leishmania* infections. Parasite loads were graded according to [23] as light (< 100 parasites per gut), moderate (100 to 1000 parasites per gut) and heavy (> 1000 parasites per gut). The experiments were repeated two times using the same vector/strain combinations. Data were evaluated statistically by means of the Fisher’s exact or Chi-square (*χ* ^2^) tests using SPSS statistics version 23 software.

### Morphometry

Parasite smears from midguts of the three sand fly species infected with *Le. amazonensis* strains were obtained on days 1 and 5–6 PI. The midguts were carefully dissected using fine needles and each part was separated and respective parasites counted. Slides were fixed with methanol, stained with Giemsa, examined under a light microscope with an oil-immersion objective and photographed with an Olympus D70 camera. For morphometry, body length, body width and flagellar length of 240 randomly selected promastigotes from six midgut smears were measured for each sand fly species and time interval using Image-J software. The morphological forms were distinguished based on the criteria of Sádlová et al. [34] and Rogers et al. [35]: (i) short nectomonads: body length < 14 μm and flagellar length < 2 times body length; (ii) long nectomonads: body length ≥ 14 μm; (iii) metacyclic promastigotes: body length < 14 μm and flagellar length ≥ 2 times body length. Data were evaluated statistically by analysis of variance using SPSS statistics version 23 software.

## Results

### Characterization of LPG repeats units

**Fig. 2:**
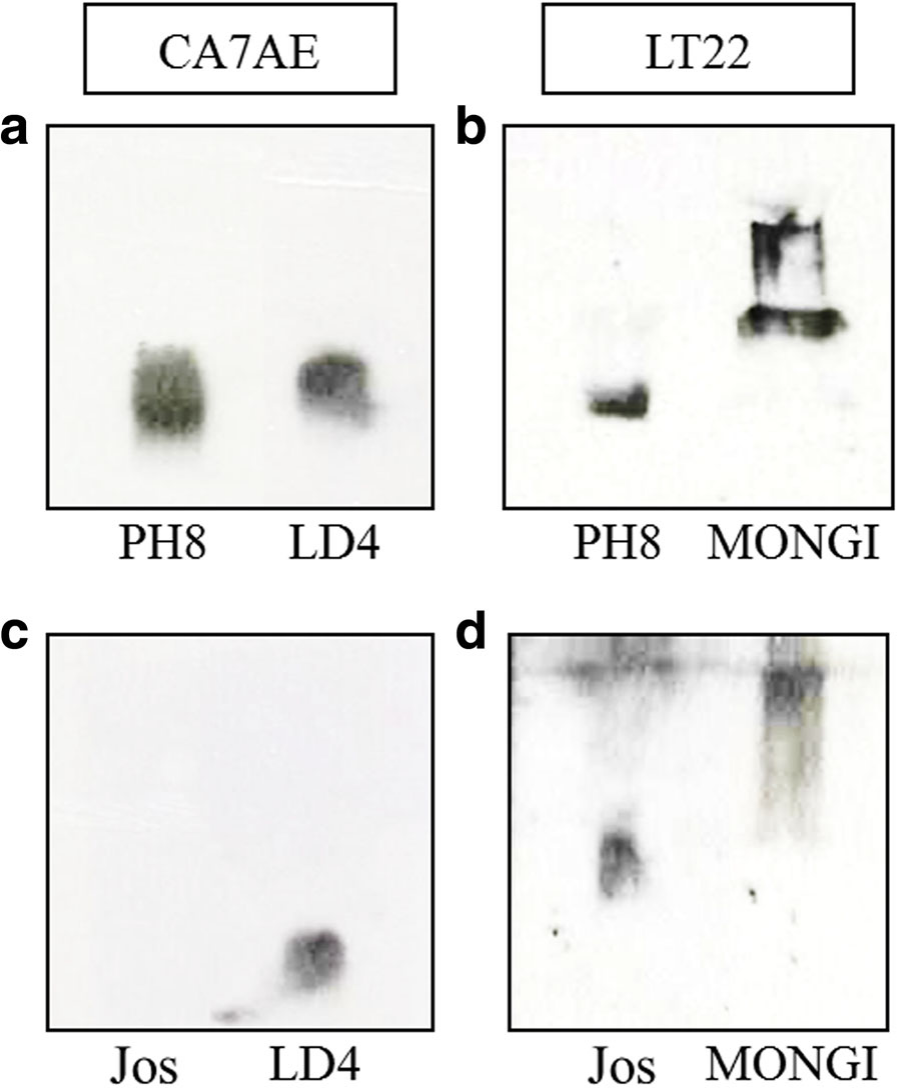
Immunoblotting of purified lipophosphoglycan (LPG). Purified LPG (10 μg per lane) from promastigotes of *Le. amazonensis* PH8 (**a** and **b**) and Josefa (Jos) (**c** and **d**) strains were incubated with the antibody CA7AE (1:1000) (**a** and **c**) and LT22 (1:1000) (**b** and **d**). The LPGs purified from *Le. donovani* LD4 and Mongi strains were used as positive controls

Purified LPGs from *Le. amazonensis* strains were differentially recognized by the mAbs CA7AE and LT22 (Fig. 2a-d). As shown in Fig. 2a-b, the LPG from the PH8 strain and respective controls (LD4 and Mongi) were recognized by CA7AE and LT22. However, a different recognition profile was observed for the Josefa strain since its LPG was recognized by LT22 (Fig. 2d) but not by CA7AE (Fig. 2c), indicating the presence of side-chains branching-off the repeat units.

**Fig. 3:**
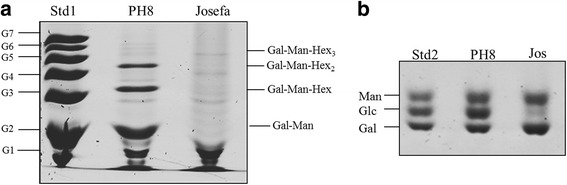
Fluorophore-assisted carbohydrate electrophoresis (FACE) of lipophosphoglycan (LPG) repeat units and monosaccharides of *Le. amazonensis* (PH8 and Josefa strains). **a** FACE analysis of dephosphorylated repeat units of PH8 and Josefa strain. Lane Std1: oligoglucose ladder represented by G_1_-G_7_; Lane PH8: repeat unit of PH8 strain; Lane Josefa: repeat unit of Josefa strain. **b** FACE of monosaccharides from LPG repeat units after strong acid hydrolysis. Lane Std2: monosaccharide standards (Man = mannose, Glc = glucose, Gal = galactose); Lane PH8: PH8 strain; Lane Jos: Josefa strain

Confirming the previous immunoblotting profiles, both *Le. amazonensis* strains displayed a distinct oligosaccharide profile of their neutral repeat units (Fig. 3a). The repeat units of the PH8 strain exhibited higher disaccharide content, represented by Gal-Man (G_2_ position) that is common to all LPGs, which explains their reactivity against CA7AE (Fig. 2a). The FACE analysis also revealed up to 1 and 2 side-chains in their structures (Fig. 3a). On the other hand, the repeat units of the Josefa strain showed up to 1 to 3 side-chains, and no Gal-Man disaccharide was detected (Fig. 3a). This profile elucidates the observed lack of recognition by CA7AE (Fig. 2c) and the positive reaction against LT22 (Fig. 2d), suggesting that most, if not all, repeating units contained side-chains.

The monosaccharide profile of the PH8 strain revealed galactose and mannose, common to all LPGs, and high content of glucose (Fig. 3b). These data confirmed the presence of glucose as side-chains, as previously indicated by immunoblotting. The Josefa strain, however, exhibited only galactose and mannose as monosaccharides (Fig. 3b).

**Fig. 4:**
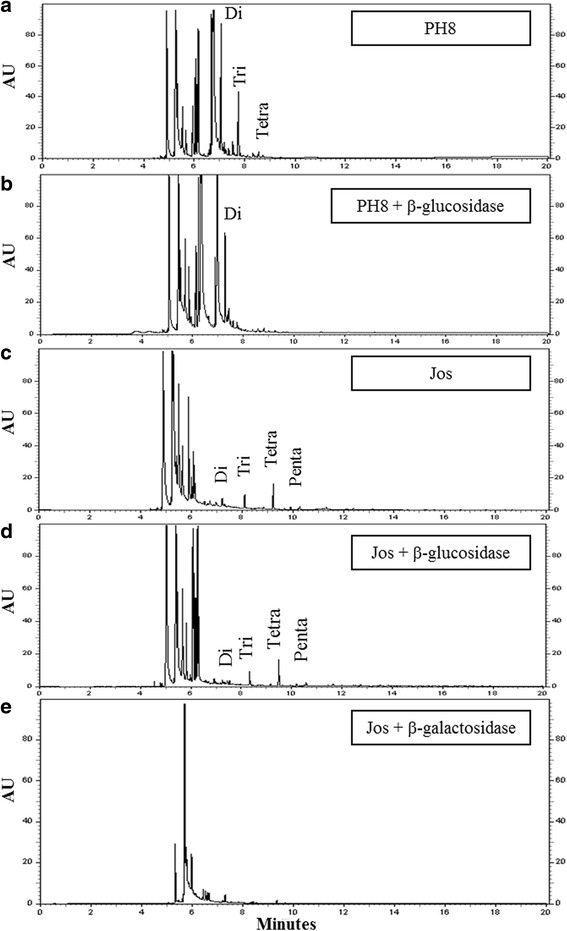
Capillary electrophoresis (CE) analyses of dephosphorylated LPG repeat units from *Le. amazonensis* strains (PH8 and Josefa). Designations above the peaks are retention times in minutes as well as di, disaccharide; tri, trisaccharide, etc. **a** LPG PH8 strain repeat units. **b** LPG PH8 strain treated with β-glucosidase. **c** LPG Josefa strain repeat units. **d** LPG Josefa strain treated with β-glucosidase. **e** LPG Josefa strain treated with β-galactosidase

**Fig. 5:**
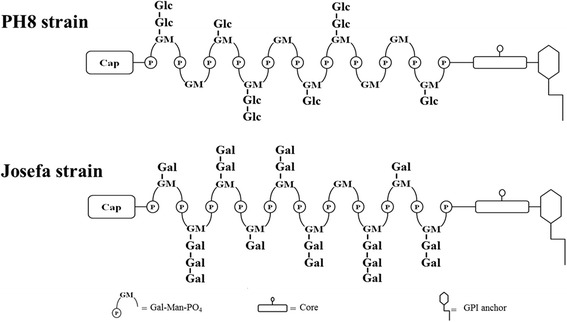
Schematic diagram of *Le. amazonensis* LPG structures of promastigotes from PH8 and Josefa strains. The repeat units contain the Gal(β1,4)Man(α1)-PO_4_ as backbone structure. For PH8 strain, the LPG bears sugar branch substitutions of one or two β-glucose residues, while in Josefa strain most of the side chains are terminated in β-galactoses. The precise locations of the sugar side chains in the repeat unit domain are not known

Confirming the FACE analysis, the dephosphorylated repeat units from the PH8 strain consisted of a di- (67%), a tri- (30%) and a tetrasaccharide (3%) (Fig. 4a). The trisaccharide and tetrasaccharide were susceptible to treatment with β-glucosidase (Fig. 4b). These data are consistent with the structure of the trisaccharide as Glc(β)Gal(β1,4)Man and the tetrasaccharide as Glc_2_(β)Gal(β1,4)Man. The LPG of the Josefa strain showed a small amount of di- (9%), an abundance of tri- (29%) and tetra- (57%), and again a small amount of pentasaccharide (5%) (Fig. 4c). The minimal disaccharide content supports the non-reactivity by CA7AE (Fig. 2c). Interestingly, this LPG was not susceptible to β-glucosidase treatment (Fig. 4d), indicating that another sugar is terminating the repeat units. After β-galactosidase treatment, we observed the disappearance of side-chains confirming the presence of β-galactoses (Fig. 4e). These results confirmed that both strains possess intraspecific polymorphisms (Fig. 5).

### Sand fly infections and morphometry

**Fig. 6:**
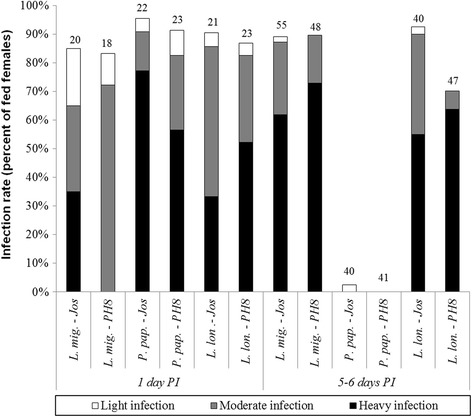
Experimental infections of *Le. amazonensis* (PH8 and Josefa strains) in *Lu. longipalpis* (*L. lon.*), *Lu. migonei* (*L. mig.*) and *Ph. papatasi* (*P. pap.*). Rates and intensities of infections were evaluated microscopically on days 1 and 5–6 post-infection (PI), and were classified into three categories: light (< 100 parasites/gut), moderate (100–1000 parasites/gut), or heavy (> 1000 parasites/gut). Data are representative of two experiments

**Fig. 7:**
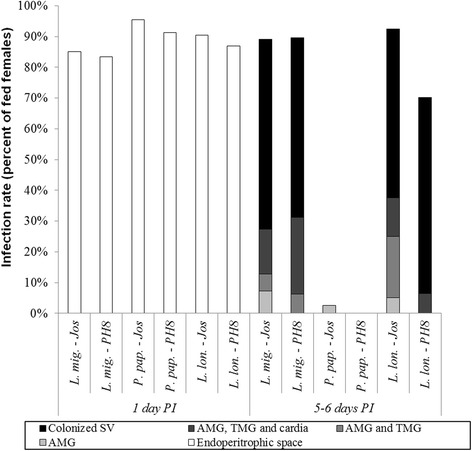
Localization of *Le. amazonensis* (PH8 and Josefa strains) in *Lu. longipalpis* (*L. lon.*), *Lu. migonei* (*L. mig.*) and *Ph. papatasi* (*P. pap.*). Localization of infections in vectors (stomodeal valve, SV; abdominal midgut, AMG; thoracic midgut, TMG; cardia and endoperithrophic space) was evaluated microscopically on days 1 and 5–6 post-infection (PI). Data are representative of two experiments

The development of *Le. amazonensis* strains was studied in *Lu. migonei*, *Lu. longipalpis* and *Ph. papatasi* on days 1 and 5–6 PI. During the early phase of infection (on day 1 PI), the infection rates were fully comparable in all parasite-vector combinations; more than 82% of females were infected and moderate or heavy intensities of infections were observed in majority of females. Parasites were in the endoperitrophic space within the blood meal surrounded by the PM. On days 5–6 PI, both *Le. amazonensis* strains could establish heavy late stage infections with the colonization of the stomodeal valve in permissive vectors *Lu. longipalpis* and *Lu. migonei*. In contrast, in *Ph. papatasi* the infections were lost during defecation of bloodmeal remnants (Figs. 6 and 7).

**Fig. 8:**
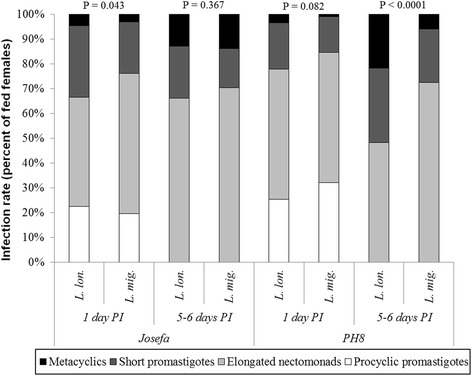
Morphological forms (metacyclics, short promastigotes, elongated nectomonads and procyclic promatigotes) of *Le. amazonensis* (PH8 and Josefa strains) during development in *Lu. longipalpis* (*L. lon.*) and *Lu. migonei* (*L. mig.*). Morphological forms of *Leishmania* parasites in vectors were evaluated microscopically on days 1 and 5–6 post-infection (PI). Data are representative of two experiments and were evaluated statistically by analysis of variance. *P* < 0.05 was considered statistically significant

Since no infection was developed in *Ph. papatasi* after defecation, morphological analysis was performed on *Le. amazonensis* parasites derived from *Lu. migonei* and *Lu. longipalpis* infections on days 1 and 5–6 PI. Both strains accomplished metacyclogenesis in both vectors. Interestingly, *Lu. longipalpis* infections with PH8 strains resulted in a higher percentage of metacyclic forms compared to *Lu. migonei* by days 5–6 PI (*P* < 0.0001) (Fig. 8).

## Discussion

*Leishmania amazonensis*, a member of the *Leishmania mexicana* complex, is one of the most studied *Leishmania* species. It is an excellent model for immunology, molecular biology and chemotherapy. This species is often associated with treatment failure, being naturally resistant to the first line chemotherapy drugs [36]. However, studies involving *Le. amazonensis* glycoconjugates and their interaction with sand fly vectors are still scarce.

The LPGs are implicated in a variety of functions in the sand flies including attachment to a microvilli receptor in Old World *Ph. papatasi* (restrictive) [37]. Early in vitro studies reported the role of interspecies LPG polymorphisms in the specificity during sand fly-*Leishmania* interactions [4]. In this study, *Ph. papatasi* midguts were recognized only by *Le. major* phosphoglycan (PG), whereas those from *Ph. argentipes* (permissive) were recognized by PGs from several species. Later, several in vitro studies demonstrated that those models are consistent while using natural vector-*Leishmania* pairs not only in Old World but also in New World vectors. For example, the interaction with PGs from New World *Le. infantum* and *Le. braziliensis* adversely affected parasite attachment in permissive *Lu. longipalpis* and restrictive *Lutzomyia intermedia*/*Lu. whitmani* [10, 38–40]. However, in vitro system limitations appear while using unnatural sand fly-*Leishmania* combinations including the attachment of *Le. braziliensis* (New World) promastigotes to *Ph. papatasi* (Old World) midguts [41]. To circumvent such limitations, in this study we have performed experiments using in vivo models with previously tested permissive sand flies known to become infected with *Le. amazonensis* [12, 19, 20].

In the New World species of *Leishmania*, LPG variations were only studied in *Le. infantum*. In this species, polymorphisms in the glucose levels were not important for in vivo infectivity to *Lu. longipalpis* [11]. However, this sugar was important for in vitro interaction of *Le. infantum* (PP75 strain) with the midguts of *Lu. longipalpis* [10].

Consistent with our previous observations, a more detailed biochemical analysis of LPG using FACE and CE from *Le. amazonensis* strains revealed important intraspecific polymorphisms. The LPG of the PH8 strain displayed β-glucose residues as side-chains whereas the Josefa strain had β-galactose residues (Fig. 5). The β-glucose residues are commonly found in the New World species of *Leishmania* including *Le. mexicana* [42], *Le. infantum* [10] and *Le. braziliensis* [31]. Those β-glucose residues in *Le. infantum* procyclic were downregulated after metacyclogenesis resulting in loss of in vitro interaction with the midgut of *Lu. longipalpis* [10]. Surprisingly, the occurrence of β-galactose residues as side-chains in the Josefa strain of *Le. amazonensis* was detected for the first time in a New World *Leishmania* species. This sugar is often observed in the Old World *Leishmania* species including *Le. major* [15], *Le. tropica /Leishmania aethiopica* [5, 7] and *Le. turanica* [17]. Unlike *Le. major* and *Le. turanica*, *Le. amazonensis* Josefa strain with β-galatosylated LPG could not survive inside *Ph. papatasi*. This phenomenon could be explained by other LPG-independent mechanisms, probably related to the distance of the sand fly-*Leishmania* pair used (Old *vs* New World) or other molecules involved. Together with LPG, glycoprotein 63 (GP63) was also evaluated in *Le. mexicana/Le. amazonensis* - *Lu. longipalpis* attachment in vivo [43] or in vitro [44]. A recent study demonstrated that both glycoconjugates could be determinant for inhibiting in vitro parasite attachment in *Lu. longipalpis* and *Lu. intermedia* [40].

Several studies have already described the differentiation of *Le. amazonensis*, *Le. braziliensis* and *Le. infantum* in New World sand fly species including *Lu. longipalpis* and *Lu. migonei* [12, 19, 20, 45]. Consistent with those observations, both *Le. amazonensis* strains could survive defecation of permissive vectors *Lu. longipalpis* and *Lu. migonei*, establish late-stage infections, and colonize anterior midgut reaching the stomodeal valve. This is a strong indication that the parasite could attach and migrate towards the mouth parts for subsequent transmission. More importantly, both strains accomplished metacyclogenesis. This morphological transformation was more pronounced in *Lu. longipalpis* infected with the PH8 strain, probably due to the higher permissiveness of this species. This sand fly can sustain a wide variety of pathogens, including viruses, non-*Leishmania* protozoans and even helminths (reviewed in [18]).

## Conclusions

In combination with previous studies, the LPG polymorphism in *Le. amazonensis* did not affect infection of the three sand fly species tested. To our knowledge, the biochemical data obtained from the Josefa strain represents the first description of a galactosylated LPG in a New World *Leishmania* species. However, these residues were not sufficient for survival in *Ph. papatasi*.

## Abbreviations

ADCL: Anergic diffuse cutaneous leishmaniasis
CE: Capillary electrophoresis
FACE: Fluorophore-assisted carbohydrate electrophoresis
GPI: Glycosylphosphatidylinositol
LCL: Localized cutaneous leishmaniasis
LPG: Lipophosphoglycan
PI: Post-infection
